# Case report and literature review: exploration of molecular therapeutic targets in recurrent malignant meningioma through comprehensive genetic analysis with Todai OncoPanel

**DOI:** 10.3389/fneur.2023.1270046

**Published:** 2023-11-23

**Authors:** Kenta Ohara, Satoru Miyawaki, Hirofumi Nakatomi, Atsushi Okano, Yu Teranishi, Yuki Shinya, Daiichiro Ishigami, Hiroki Hongo, Shunsaku Takayanagi, Shota Tanaka, Aya Shinozaki-Ushiku, Shinji Kohsaka, Hidenori Kage, Katsutoshi Oda, Kiyoshi Miyagawa, Hiroyuki Aburatani, Hiroyuki Mano, Kenji Tatsuno, Nobuhito Saito

**Affiliations:** ^1^Department of Neurosurgery, Faculty of Medicine, The University of Tokyo, Tokyo, Japan; ^2^Department of Pathology, The University of Tokyo Hospital, Tokyo, Japan; ^3^Division of Cellular Signaling, National Cancer Center Research Institute, Tokyo, Japan; ^4^Department of Next-Generation Precision Medicine Development Laboratory, Graduate School of Medicine, The University of Tokyo, Tokyo, Japan; ^5^Division of Integrative Genomics, Graduate School of Medicine, The University of Tokyo, Tokyo, Japan; ^6^Laboratory of Molecular Radiology, Center for Disease Biology and Integrative Medicine, Graduate School of Medicine, The University of Tokyo, Tokyo, Japan; ^7^Genome Science and Medicine Laboratory, Research Center for Advanced Science and Technology, The University of Tokyo, Tokyo, Japan

**Keywords:** malignant meningioma, malignant progression, Todai OncoPanel, comprehensive genomic analysis, actionable gene aberration

## Abstract

**Background:**

Despite accumulating research on the molecular characteristics of meningiomas, no definitive molecularly targeted therapy for these tumors has been established to date. Molecular mechanisms underlying meningioma progression also remain unclear. Comprehensive genetic testing approaches can reveal actionable gene aberrations in meningiomas. However, there is still limited information on whether profiling the molecular status of subsequent recurrent meningiomas could influence the choice of molecular-targeted therapies.

**Case presentation:**

We report a case of meningioma with malignant progression and multiple recurrences. We performed matched tumor pair analysis using the Todai OncoPanel to investigate the possibility of additional standard treatments. The loss of several chromosomal regions, including *NF2* and *CDKN2A*, which is associated with aggressive meningiomas, was considered a significant driver event for malignant progression. Using additional matched tumor pair analysis, mutations in *TRAF7, ARID1A*, and *ERBB3* were identified as subclonal driver events at the time of recurrence. No genetic aberrations were found for which evidence-based targeted therapy was applicable. We also reviewed previous reports of molecular therapies in meningioma to discuss issues with the current molecular testing approach.

**Conclusion:**

Gene panel testing platforms such as the Todai OncoPanel represent a powerful approach to elucidate actionable genetic alterations in various types of tumors, although their use is still limited to the diagnosis and prediction of prognosis in meningiomas. To enable targeted molecular therapy informed by gene-panel testing, further studies including matched tumor pair analyses are required to understand the molecular characteristics of meningiomas and develop treatments based on genetic abnormalities.

## 1 Introduction

The treatment of malignant meningioma remains challenging due to the absence of alternatives other than maximum surgical removal and radiation therapy (1). With recent advances in next-generation sequencing, several molecular approaches have been developed to understand the molecular characteristics of meningiomas. In addition to the well-known deletion of chromosome 22 and mutation of *NF2* (2–5), other driver gene mutations in *TRAF7*, *KLF4*, *AKT1*, *SMO*, and *POLR2A* have also been identified (6–10). Furthermore, DNA methylation and gene expression profiles have been studied in meningioma (11–14). Several molecularly targeted therapies for meningiomas have been attempted based on alterations identified in specific genes or their associated signaling pathways. Although some therapies are potentially effective (15–20), a definitive treatment has not yet been established. As reports analyzing acquired molecular aberrations with recurrent paired specimens have been limited (21, 22), molecular mechanisms underlying meningioma progression are still unclear.

Fortunately, large-scale genomic sequencing has identified numerous actionable gene aberrations in various tumor types (23–26). We have clinically applied the Todai OncoPanel (TOP) for the detection of cancer-related genes at our institution (26). This panel is characterized by a twin-panel system incorporating DNA and RNA that is effective in detecting fusion transcripts (26–28). However, the clinical utility of these panel tests for central nervous system tumors remains limited (29).

Here, we report a case of refractory malignant meningioma that was evaluated by comprehensive molecular testing to explore the potential indications for new targeted therapies. We focus on whether changes in the molecular profiles of matched recurrent meningiomas could influence the choice of molecular-targeted therapies. To better understand therapeutic approaches in meningiomas, this study reviewed the relevant literature or ongoing clinical trials based on potential therapeutic targets. We also discuss its usefulness and future issues associated with clinical panel sequencing in meningioma treatment.

## 2 Case description

**Figure 1:**
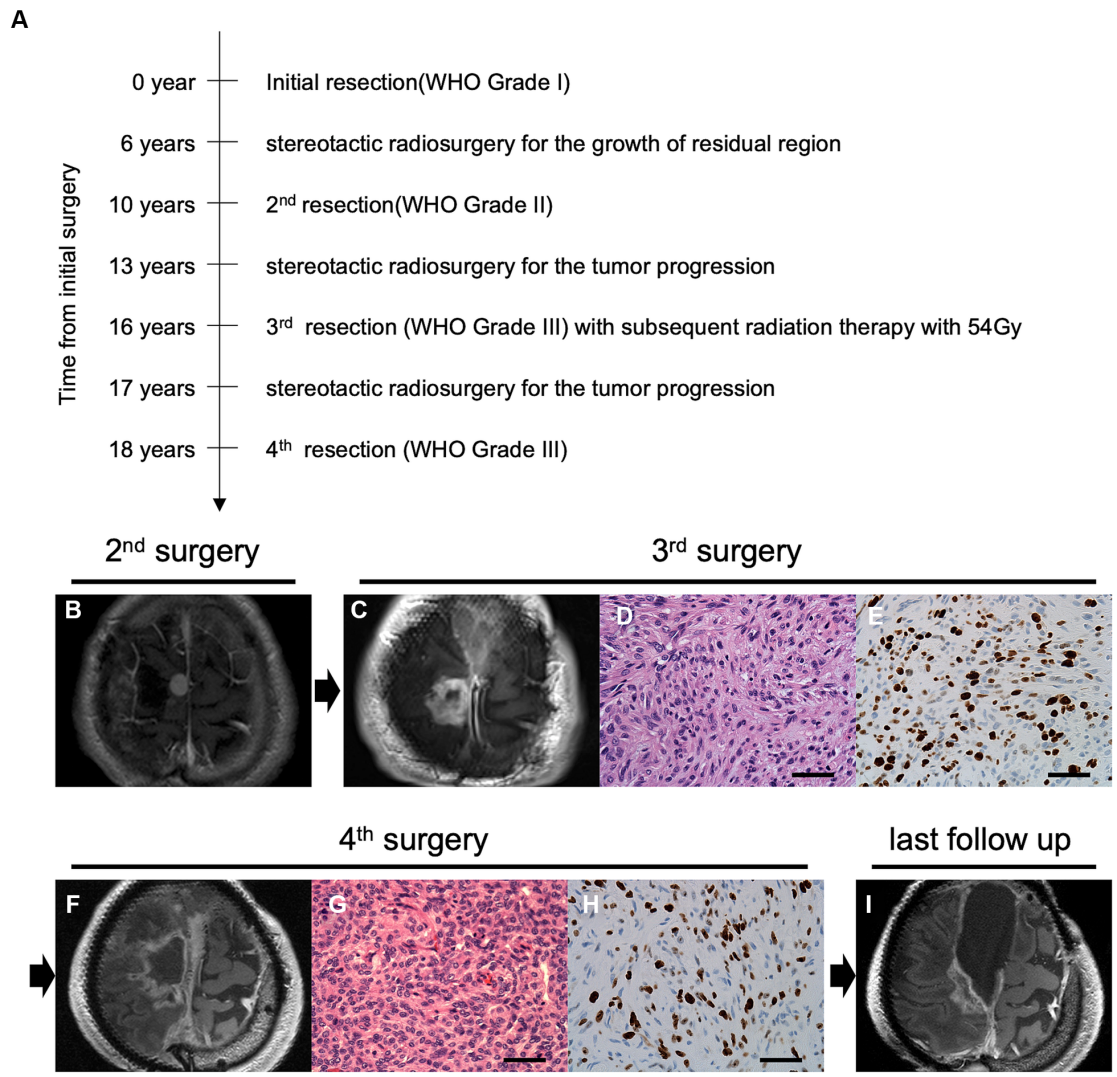
Time course and clinical findings of the progressive meningioma. **(A)** The diagram shows the time course of treatment and tumor progression. Preoperative magnetic resonance imaging (MRI) using gadolinium-enhanced T1 imaging (Gd-T1WI) of the parasagittal sinus meningioma at the second **(B)** and third **(C)** surgeries. Pathological features of the tumor at the third surgery indicate overt anaplasia by malignant progression with hematoxylin and eosin (H&E) staining **(D)** and high mitotic features in Ki-67 staining **(E)** under ×400 magnification (scale bar = 50 μm). Preoperative Gd-T1WI before the fourth tumor removal **(F)**. H&E staining **(G)** and Ki67 staining **(H)** at the fourth surgery (original magnification, ×400; scale bar = 50 μm). Postoperative follow-up imaging using Gd-T1WI shows tumor progression in the posterior cavity **(I)**.

A 55-year-old man had undergone initial tumor resection for a parasagittal meningioma, defined as World Health Organization (WHO) grade 1, at another hospital (Figure 1A). He had no significant medical history or family history of meningioma. After gamma knife radiosurgery for recurrence at the age of 61 years, a second resection had been performed at 65 years of age due to progressive tumor growth with histological transformation to a WHO grade 2 atypical meningioma (Figure 1B). At 68 years of age, he had been treated again with stereotactic radiosurgery for local recurrence. Due to tumor regrowth, he was referred to our hospital for a third surgery at 71 years of age (Figure 1C). On preoperative physical examination, he showed mild paralysis of the right lower limb. Manual muscle testing (MMT) of the right lower limb showed grade 4. His postoperative course was uneventful. The pathological specimen of the tumor indicated a diagnosis of malignant progression to anaplastic meningioma, WHO grade 3, with overt anaplasia and a high Ki-67 index (Figures 1D,E). Postoperative adjuvant radiation therapy was administered at a dose of 54 Gy. Two years later, a fourth surgical resection was required with progressive gait disturbance, and the patient was again diagnosed with an anaplastic meningioma (Figures 1F–H). After the surgery, his paralysis of the right lower limb worsened to MMT grade 3. With rehabilitation, his paralysis improved MMT grade4. He was able to walk with a cane and lead a largely independent life. Another recurrent lesion progressed toward the eloquent motor area at the posterior aspect of the tumor removal cavity (Figure 1I); however, surgical resection of the lesion was associated with a high risk of postoperative paralysis, and additional radiotherapy was ineffective. Considering that standard therapies were not viable, the patient wanted to explore the possibility of targeted molecular therapy. After thoroughly explaining that discovering a new treatment for meningioma with our panel analysis has yet to be established, he requested our genetic testing. Therefore, we performed comprehensive panel testing to elucidate whether this refractory meningioma possesses actionable gene aberrations suitable for targeted molecular therapies.

### Todai OncoPanel analysis

We conducted comprehensive panel sequencing using TOP after obtaining the appropriate informed consent from the patient. The study was performed in accordance with the principles of the Declaration of Helsinki and was approved by the ethics committee of the University of Tokyo. The method of analysis has been reported previously (26). Briefly, this unique custom-made panel includes DNA and RNA components. The TOP DNA panel targets 464 genes to detect single-nucleotide variants (SNVs), small insertions/deletions, and copy number variations (CNVs). The TOP RNA panel detects 463 fusion genes using the junction capture method. In addition, various probes detect single nucleotide polymorphisms. By comparing tumor and normal reads, chromosomal gains and losses are visualized as a chromosomal copy number graph (Supplementary Tables 1, 2). The tumor resected in the fourth surgery (Tumor S4) was mainly used to detect actionable gene aberrations, whereas the tumor resected in the third surgery (Tumor S3) was used for comparisons with Tumor S4. The detected genetic and transcriptional alterations were reviewed and classified according to the level of evidence and potential treatments by an expert panel consisting of physicians, pathologists, genetic counselors, molecular biologists, and cancer genome researchers.

### 2.2 Genetic findings

**Figure 2:**
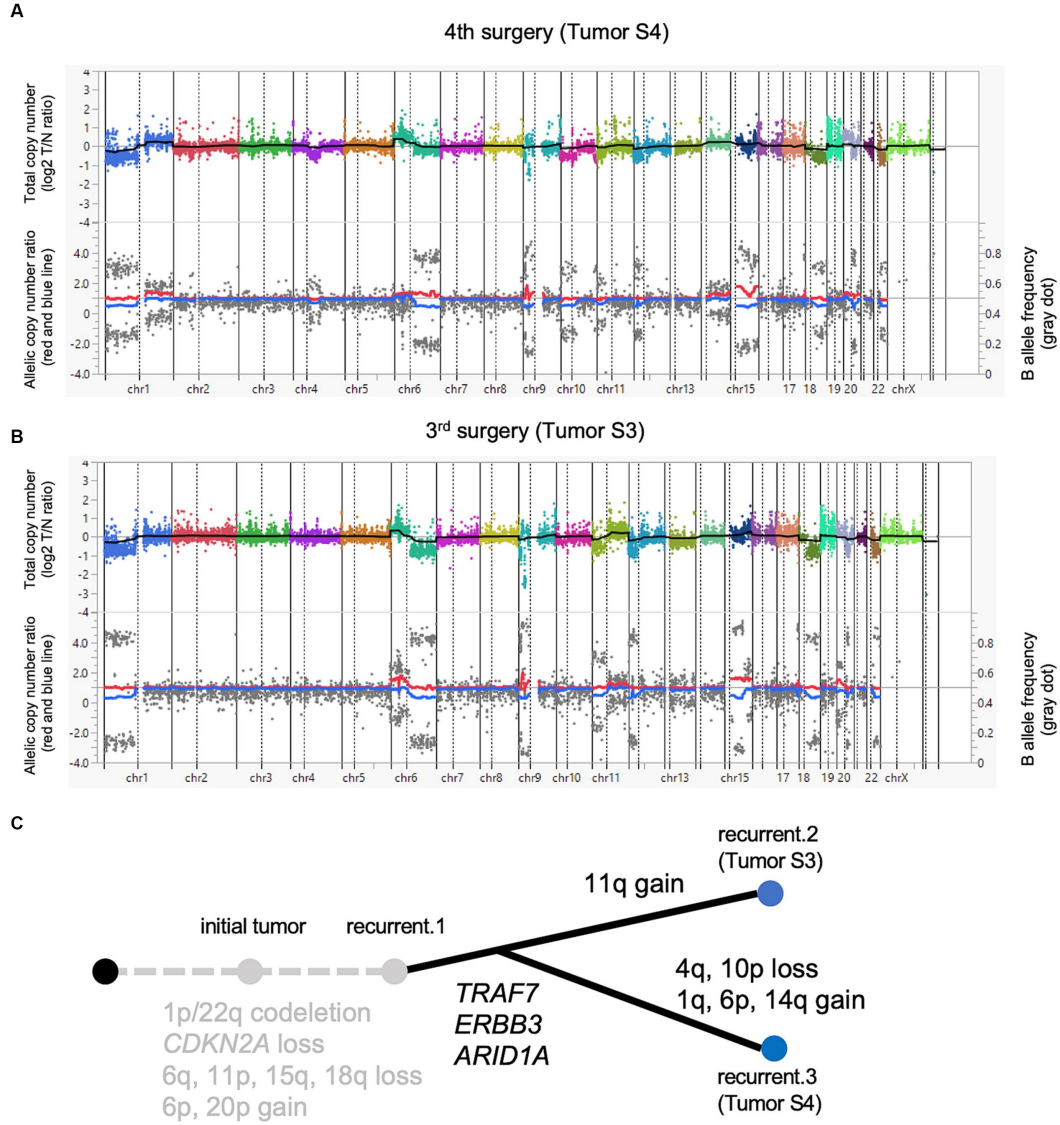
A paired analysis with Todai OncoPanel. Chromosomal copy number variations in anaplastic meningioma. The upper panel shows the total copy number, and the lower panel shows the allelic copy number ratio with B allele frequency at the fourth **(A)** and third surgeries **(B)**. Implications for driver events along with tumor progression **(C)**.

Both tumors were sequenced at a high depth in the TOP DNA panel (mean depth: 1196.4× for Tumor S3 and 1390.5× for Tumor S4). No significant difference was found in tumor purity (53.0% for Tumor S3 and 55.0% for Tumor S4, respectively; data not shown). Also, tumor cell compositions were similar in both histopathological images. We identified five non-synonymous mutations and one splice-site mutation in Tumor S4, with a detection threshold of variant allele frequency (VAF) > 5% (Table 1). The *TRAF7* mutation c.1168G > A (p.Gly390Arg), a frequent mutational hotspot in meningiomas, was detected. *ARID1A*, a component of the SWI/SNF complex that acts as a driver in high-grade meningiomas, was also mutated. We also found multiple chromosomal copy number losses, including 1p/22q co-deletion (Figure 2A). A 1q gain, which is associated with poor outcomes in meningiomas, was also observed. In addition, we identified various genetic CNVs, including *CDKN2A* deletions (Supplementary Table 3). TOP RNA testing revealed no fusion transcripts. We could not identify actionable gene aberrations that could be potential targets of approved drugs or clinical trials in expert panel reviews.

**Table 1:** Tumor genetic variants identified using Todai OncoPanel.

Gene	CytoBand	Variant	Amino acid	Mutation type	VAF	
					Tumor S4	Tumor S3
*TRAF7*	16p13.3	c.1168G > A	p.G390R	Missense	28.8%	1.3%
*ARID1A*	1p36.11	c.1048 T > G	p.S350A	Missense	5.6%	3.3%
*ERBB3*	12q13.2	c.2938-38G > T	-	Splice-site	22.8%	1.9%
*ERBB3*	12q13.2	c.2954G > A	p.G985E	Missense	24.6%	1.5%
*ERBB3*	12q13.2	c.3010G > A	p.E1004K	Missense	26.2%	1.6%
*ERBB3*	12q13.2	c.3016G > A	p.E1006K	Missense	27.2%	1.8%
*ERCC2*	19q13.32	c.1034del	p.R345Lfs*14	Frameshift	Undetected	9.2%

VAF, Variant allele frequency.

Next, we compared the genomic abnormalities of Tumor S3 with Tumor S4 to explore the differences that emerged during tumor progression. Tumor S4 showed six non-synonymous mutations with a VAF > 5%, whereas these mutations were detected with VAF less than 5% in Tumor S3. One mutation that was not detected in Tumor S4 was detected in S3 with VAF > 5% (Table 1). Genetic CNVs, including those in *CDKN2A*, were partially shared throughout tumor progression without notable changes (Supplementary Table 3). Although the profile of the chromosomal CNVs of Tumor S3 was similar to that of Tumor S4, some chromosomal changes, such as the gain of 1q, 6p, and 14q and the loss of 4q and 10p, were additionally observed in Tumor S4 (Figure 2B), suggesting that Tumor S4 exhibits a pattern of branched clonal evolution from Tumor S3 (Figure 2C). Unfortunately, as no suitable molecularly targeted therapeutic agent exists for the patient’s course, conservative follow-up was continued despite the continuously growing tumor.

## 3 Literature review of molecular targeted therapies for meningiomas

A search strategy was conducted according to the Preferred Reporting Items for Systematic Reviews and Meta-Analyses (PRISMA) guidelines (30). We searched using the term:(“meningioma”[MeSH] AND “drug therapy”) to identify relevant articles in MEDLINE^1^ up to September 2023. We included articles that were original prospective phase II trials of molecular targeted therapies for meningiomas to demonstrate options for potentially applicable treatment in the future. To avoid missing relevant research efforts, we also hand-searched other articles on Phase II trials. Next, we searched for ongoing clinical trials for meningiomas on ClinicalTrials.gov to September 2023. We included ongoing phase II or III trials that focused only on meningioma.

## 4 Discussion

Here, we report a case of progressive meningioma that was evaluated by molecular profiling. Contrary to our expectations, no actionable genetic aberrations were detected. However, we obtained some implicative results via a genetic analysis of paired recurrent samples. In this progressive case, we identified the *TRAF7* mutation, which is typically found in benign meningiomas. Although this mutation was detected in both S3 and S4, the VAF of S3 was markedly low without differences in tumor purity. Tumor heterogeneity may have influenced the results, but this mutation may have been acquired as a subclonal driver event. *TRAF7* mutations are often associated with mutations in other genes, such as *AKT1*, *KLF4*, and *PIK3CA* (8, 9), and rarely with *NF2* alterations, suggesting that *TRAF7* mutation may not represent the earliest driver event, as in this case. Regarding the significance of the “add-on” *TRAF7* mutations, the accumulation of matched-pair analysis using recurrent specimens may help confirm this hypothesis.

Considering that the TOP test targeted sufficiently large genetic regions, we also identified *NF2* inactivation and chromosomal abnormalities, such as the loss of 1p, 6q, 10p, and 18q and deletion of *CDKN2A*, which indicated tumor aggressiveness in the present case (21, 31, 32). Interestingly, the 1q gain, which is harbored in the most aggressive types of meningioma, was acquired in Tumor S4 (33). High-grade meningiomas frequently exhibit *NF2* alterations (6, 8, 9). Furthermore, the number of genetic and chromosomal CNVs indicates the risk of recurrence and aggressiveness in malignant meningiomas and even a subset of benign WHO grade 1 tumors (22, 34). Although a variety of driver genetic events can be detected in a single genetic panel test in meningiomas (29, 35, 36), CNV analysis is also required to predict meningioma aggressiveness. Some reports have shown the usefulness of CNV analysis using DNA panel tests for meningiomas (29, 36). The behavior and recurrence risk of meningiomas are generally difficult to predict based on clinical features (e.g., the Simpson grading scale and WHO grading system) (37). Therefore, TOP analysis offers a significant advantage over other diagnostic tools by revealing the genetic profiles of meningiomas and identifying tumors associated with poor prognosis.

However, panel testing shows limitations in its therapeutic application. In multiple types of tumors, targeted gene panel testing cost-effectively clarifies the genetic background and identifies targetable gene aberrations. However, an unignorable discrepancy exists between the level of identified actionable gene aberrations and that of patients receiving accordingly targeted therapies. Actionable gene aberrations of various tumors are identified in 32.2%–59.4% of patients, whereas the level of patients who receive molecularly targeted therapy remains at approximately 10% (23–26). This discrepancy may be associated with the scarcity of established molecularly targeted therapies in comparison with the number of detectable genetic abnormalities. Even if a potentially effective therapeutic agent exists, the treatment cannot be administered without prior clinical validation. The presence of actionable gene mutations varies depending on the tumor type. Genomic panel testing is considered applicable for tumors for which molecularly targeted drugs are already available, whereas the applicability of molecularly targeted therapy is still limited in other tumors, including intracranial tumors.

For meningiomas, which lack established molecular therapies, gene panel testing for therapeutic purposes remains challenging without the development of novel therapeutic agents. To our knowledge, several prospective studies have demonstrated the effects of molecular targeted therapies for meningiomas (Table 2). In the previous study, targeted agents such as anti-angiogenic inhibitors, mTOR inhibitors, and EGFR inhibitors were investigated based on the activation of intracellular signaling pathways in meningiomas (15, 17–20, 38, 39, 42). Also, other clinical trials based on potential targets in meningiomas are in progress. As major genetic drivers specific to meningiomas, *NF2*, *AKT1*, and *SMO* mutations could be targeted by FAK, AKT1, and SMO inhibitors, respectively (43, 44). As an immunotherapy, PD-1 inhibitor showed promising efficacy for immunosuppressive tumor microenvironment of high-grade meningiomas (40, 41). Previous large-scale studies have suggested the therapeutic potential of CDK inhibitors and histone deacetylase inhibitors in molecularly aggressive types of meningiomas (33, 45).

**Table 2:** Review of molecular targeted therapy for meningiomas.

Previous studies of molecular targeted therapy for meningiomas								
References	*n*	WHO grade (*n*)	Intervention	Drug class	Molecular target	Phase	Radiographic response	6 M-PFS
Wen et al. (15)	23	1 (13)	Imatinib	PDGFR inhibitor	PDGFR	2	SD: 47.4%	29.40%
		2 (5)						
		3 (5)						
Norden et al. (19)	25	1 (8)	Gefitinib/erlotinib	EGFR inhibitor	EGFR	2	SD: 32%	28%
		2 (9)						
		3 (8)						
Reardon et al. (38)	21	1 (8)	Imatinib	PDGFR inhibitor	PDGFR	2	SD: 66.7%	61.90%
		2 (9)	Hydroxyurea					
		3 (4)						
Raizer et al. (18)	25	1 (2)	Vatalanib	VEGFR inhibitor	VEGFR	2	SD: 68.2%	54.40%
		2 (14)						
		3 (8)						
Kaley et al. (17)	36	1 (4)	Sunitinib	Tyrosine kinase inhibitor	VEGFR, PDGFR, KIT	2	CR/PR: 5.6% SD: 69.4%	42%
		2 (30)						
		3 (6)						
Shih et al. (39)	17	1 (4)	Everolimus	mTOR inhibitor	mTOR	2	SD: 88%	69%
		2 (7)	Bevacizumab	VEGF inhibitor	VEGF			
		3 (5)						
Graillon et al. (20)	20	1 (2)	Everolimus	mTOR inhibitor	mTOR	2	N/A	55%
		2 (10)	Octreotide					
		3 (8)						
Brastianos et al. (40)	25		Pembrolizumab	PD-1 inhibitor	PD-1	2	SD: 72%	48%
		2 (22)						
		3 (3)						
Bi et al. (41)	25	2 (18)	Nivolumab	PD-1 inhibitor	PD-1	2	PR: 4%	42.40%
		3 (7)					SD: 60%	
Kumthekar et al. (42)	42	1 (10)	Bevacizumab	VEGF binding monoclonal antibody	VEGF	2	PR:2%	Grade1:90%
		2 (21)					SD:86%	Grade2/3:66%
		3 (11)						
Brastianos et al. (43)	36	1 (12)	GSK2256098	FAK inhibitor	*NF2*	2	PR: 2.8%	50%
		2 (18)						
		3 (6)					SD: 66.7%	

Ongoing clinical trials of molecular targeted therapy for meningiomas							
NCT number	*n*	WHO grade(*n*)	Intervention	Drug class	Molecular target	Phase	Primary outcome
3071874	25	2, 3	Vistusertib	mTOR inhibitor	mTOR	2	PFS
5425004	24	2, 3	Cabozantinib	VEGF inhibitor	VEGF	2	PFS
5130866	89	-	AR-42 (OSU-HDAC42)	Histone deacetylase inhibitor	*NF2*	2, 3	PFS
2523014	124	-	Vismodeg	SMO inhibitor	*SMO*	2	PFS
			Capivasertib	AKT inhibitor	*AKT1*		
			Abemaciclib	CDK inhibitor	*CDKN2A* loss, *CDK* gain		

SD, stable disease; CR, complete response; PR, partial response; 6 M-PFS, 6-month progression-free survival; PFS, progression-free survival.

The prior studies suggest that those targeted therapies were expected to stabilize meningioma growth. However, as the results of these inhibitors are in Phase II trials, future investigations are still needed. Further, from a clinical perspective, the feasibility of these therapies is still limited because molecular testing for meningiomas is not part of routine practice. Even though well-recognized driver genetic events are not detected in some meningiomas (6–9), additional hidden molecular targets could be detected by further analysis of the increased number of these “apparently driver-negative” meningiomas.

Currently, genomic surveys with customized gene panel testing mainly contribute to personalized medicine by elucidating the genomic profile and allowing clinicians to select high-risk cases for closer follow-up. The number of analyzed cases needs to be increased to demonstrate the usefulness of TOP testing for meningiomas as a useful tool in future molecular therapy. At the same time, further molecular understanding of meningiomas and the development of therapeutic agents are required. Meningiomas show complicated diversity in their molecular landscapes, which can be identified by the integrated analysis of DNA methylation or gene expression profiles (11–14, 33, 45, 46). The correlation between molecular characteristics and specific genomic events requires elucidation. Combined panel testing such as TOP may yield comprehensive genetic profiles, including gene expression profiles, in the future. Also, matched tumor pair analysis may provide more detailed knowledge of molecular profiles.

In conclusion, gene panel analysis, including TOP, effectively elucidates various genetic alterations in meningiomas. However, panel testing is limited to diagnostic and prognostic prediction. The establishment of definitive treatments for meningiomas is essential for molecularly targeted therapy informed by genetic panel testing.

## Data availability statement

The datasets presented in this article are not readily available because of ethical and privacy restrictions. Requests to access the datasets should be directed to the corresponding author.

## Ethics statement

The studies involving humans were approved by the Institutional Review Board of the University of Tokyo. The studies were conducted in accordance with the local legislation and institutional requirements. The participants provided their written informed consent to participate in this study. Written informed consent was obtained from the individual(s) for the publication of any potentially identifiable images or data included in this article.

## Author contributions

KOh: Data curation, Writing – original draft. SM: Writing – review & editing, Supervision. HN: Data curation, Writing – review & editing. AO: Data curation, Writing – review & editing. YT: Data curation, Writing – review & editing. YS: Data curation, Writing – review & editing. DI: Data curation, Writing – review & editing. HH: Data curation, Writing – review & editing. ShuT: Data curation, Formal analysis, Writing – review & editing. ShoT: Data curation, Formal analysis, Writing – review & editing. AS-U: Data curation, Formal analysis, Validation, Writing – review & editing. SK: Formal analysis, Writing – review & editing. HK: Formal analysis, Writing – review & editing. KOd: Formal analysis, Writing – review & editing. KM: Formal analysis, Writing – review & editing. HA: Formal analysis, Writing – review & editing. HM: Formal analysis, Writing – review & editing. KT: Formal analysis, Visualization, Writing – review & editing. NS: Project administration, Supervision, Writing – review & editing.

## Footnotes

^1^ www.pubmed.gov
